# Multiparametric MRI for Prostate Cancer Characterization: Combined Use of Radiomics Model with PI-RADS and Clinical Parameters

**DOI:** 10.3390/cancers12071767

**Published:** 2020-07-02

**Authors:** Piotr Woźnicki, Niklas Westhoff, Thomas Huber, Philipp Riffel, Matthias F. Froelich, Eva Gresser, Jost von Hardenberg, Alexander Mühlberg, Maurice Stephan Michel, Stefan O. Schoenberg, Dominik Nörenberg

**Affiliations:** 1 Experimental Radiation Oncology Group, Heidelberg University, D-68167 Mannheim, Germany; piotrekwoznicki@gmail.com; 2 Department of Urology and Urosurgery, University Medical Center Mannheim, Medical Faculty Mannheim, Heidelberg University, D-68167 Mannheim, Germany; Niklas.Westhoff@medma.uni-heidelberg.de (N.W.); Jost.vonHardenberg@medma.uni-heidelberg.de (J.v.H.); Maurice-Stephan.Michel@medma.uni-heidelberg.de (M.S.M.); 3 Department of Clinical Radiology and Nuclear Medicine, University Medical Center Mannheim, Medical Faculty Mannheim, Heidelberg University, D-68167 Mannheim, Germany; Thomas.Huber@medma.uni-heidelberg.de (T.H.); Philipp.Riffel@medma.uni-heidelberg.de (P.R.); Matthias.Froelich@medma.uni-heidelberg.de (M.F.F.); stefan.schoenberg@umm.de (S.O.S.); 4 Department of Radiology, Munich University Hospitals, D-81377 Munich, Germany; Eva.Gresser@med.uni-muenchen.de; 5 Siemens Healthineers, CT R&D Image Analytics, D-91301 Forchheim, Germany; alexander-muehlberg@hotmail.com

**Keywords:** prostatic neoplasm, magnetic resonance imaging, radiomics, machine learning, artificial intelligence, PI-RADS, PSA

## Abstract

Radiomics is an emerging field of image analysis with potential applications in patient risk stratification. This study developed and evaluated machine learning models using quantitative radiomic features extracted from multiparametric magnetic resonance imaging (mpMRI) to detect and classify prostate cancer (PCa). In total, 191 patients that underwent prostatic mpMRI and combined targeted and systematic fusion biopsy were retrospectively included. Segmentations of the whole prostate glands and index lesions were performed manually in apparent diffusion coefficient (ADC) maps and T2-weighted MRI. Radiomic features were extracted from regions corresponding to the whole prostate gland and index lesion. The best performing combination of feature setup and classifier was selected to compare its predictive ability of the radiologist’s evaluation (PI-RADS), mean ADC, prostate specific antigen density (PSAD) and digital rectal examination (DRE) using receiver operating characteristic (ROC) analysis. Models were evaluated using repeated 5-fold cross-validation and a separate independent test cohort. In the test cohort, an ensemble model combining a radiomics model, with models for PI-RADS, PSAD and DRE achieved high predictive AUCs for the differentiation of (i) malignant from benign prostatic lesions (AUC = 0.889) and of (ii) clinically significant (csPCa) from clinically insignificant PCa (cisPCa) (AUC = 0.844). Our combined model was numerically superior to PI-RADS for cancer detection (AUC = 0.779; *p* = 0.054) as well as for clinical significance prediction (AUC = 0.688; *p* = 0.209) and showed a significantly better performance compared to mADC for csPCa prediction (AUC = 0.571; *p* = 0.022). In our study, radiomics accurately characterizes prostatic index lesions and shows performance comparable to radiologists for PCa characterization. Quantitative image data represent a potential biomarker, which, when combined with PI-RADS, PSAD and DRE, predicts csPCa more accurately than mADC. Prognostic machine learning models could assist in csPCa detection and patient selection for MRI-guided biopsy.

## 1. Introduction

Prostate cancer (PCa) is the second most frequent cancer diagnosis made in men, with 1,276,106 new cases and 358,989 deaths reported worldwide in 2018 [1]. Early and accurate detection of clinically significant prostate cancer (csPCa), which is defined as ISUP grade 2 or higher [2], is essential to initiate treatment in a timely manner and improve patient outcomes [3].

Over the past decades, multiparametric magnetic resonance imaging (mpMRI) and consecutive targeted biopsy have become integral diagnostic procedures for the detection and risk stratification of csPCa [4,5], showing an improvement in detection compared to systematic non-targeted prostate biopsy [5,6,7]. The Prostate Imaging-Reporting and Data System (PI-RADS) defines standards for image acquisition and reporting and is broadly utilized in clinical practice. It has recently been updated to version 2.1 to address the inconsistencies and limitations of the previously released PI-RADS versions [8]. PI-RADS v2.1 relies on the qualitative assessment of T2-weighted and diffusion-weighted (DWI), as well as dynamic contrast-enhanced (DCE) images. The sensitivity of 93% and the negative predictive value of 89% were reported for the detection of csPCa using the PI-RADS classification [3]. Prostate-specific antigen density (PSAD) has been proved to increase the specificity of prostate-specific antigen (PSA) in detecting PCa [9], however, early evidence on its use for the selection process for biopsy was conflicting. Several recent studies suggest that PSAD, combined with mpMRI, improves the negative predictive value of PI-RADS scoring [10,11]. Lesion volume, as estimated on T2w MRI, has also been independently associated with PCa detection and clinical significance [12]. Digital rectal examination (DRE) is commonly performed to screen for PCa, although a recent meta-analysis demonstrated its low efficacy with a pooled sensitivity of 0.51 and specificity of 0.59 for PCa detection [13]. To deliver the benefits of the MRI-diagnosis pathway, there is an important need to increase work efficiency while minimizing variability in MRI data acquisition and in reader interpretations and to reduce the time needed to identify men who are likely to have csPCa.

Machine learning is a field of artificial intelligence (AI) covering an extensive range of algorithms that can be trained to perform a certain task, such as classification. Supervised learning methods, which use labeled data to guide the algorithm, have arguably seen the biggest success in medical imaging, with applications including diagnosis of metastatic disease, diabetic retinopathy, or intracranial hemorrhage [14,15]. Typical supervised machine learning approaches include an end-to-end classification on input images with convolutional neural networks (CNNs) or classification on features priorly extracted from the image. 

Radiomic analysis is defined as computationally extracting quantitative image features for the characterization of disease patterns. Analyzed image features include shape features, first-order statistics, and texture characteristics, such as grey level co-occurrence matrices (GLCM) [16]. Recent advances in image analysis techniques using radiomic features to assess genomic, proteomic, and clinical phenotypes of the prostate tumor were shown to match or even surpass the qualitative imaging assessment of radiologists [17,18,19]. 

The aim of this study was to develop predictive machine learning models for prostate cancer detection and classification and compare them with the PI-RADS-based assessment of radiologists and quantitative imaging as well as clinical biomarkers using ROC analysis and specificity threshold equivalent to clinical reporting [20]. We focused on two clinically relevant classification tasks: (i) malignant vs. benign prostate lesion and (ii) csPCa vs. cisPCa classification.

## 2. Results

### 2.1. Demographic Data

Demographic and clinical characteristics, as well as MRI findings of the entire study cohort, are summarized in Table 1. The analyzed parameters did not follow normal distribution and thus, are summarized with median and IQR. Of the 191 enrolled patients, 151 patients were included in the training cohort (median age 68 years, IQR: 63–74 years, median serum PSA 7.60 ng/ml, IQR: 5.71–11.0 ng/ml) and 40 patients were included in the test cohort (median age 69 years, IQR: 63–72 years, median serum PSA 8.17 ng/ml, IQR: 6.82–11.85 ng/ml). No patients were under ongoing medical therapy. For all 191 included patients, a single index lesion per patient was segmented and scored according to PI-RADS v2.0. MR-/Ultrasound fusion biopsy of suspicious index lesions (and following standard TRUS-guided 12-core prostate biopsy) revealed 102 patients with benign lesions and 89 patients with histologically proven PCa, including 65 patients with csPCa and 24 with cisPCa, respectively. Clinically significant cancer was surgically defined as a Gleason score of 7 (ISUP grade 2) or greater.

**Figure 1:**
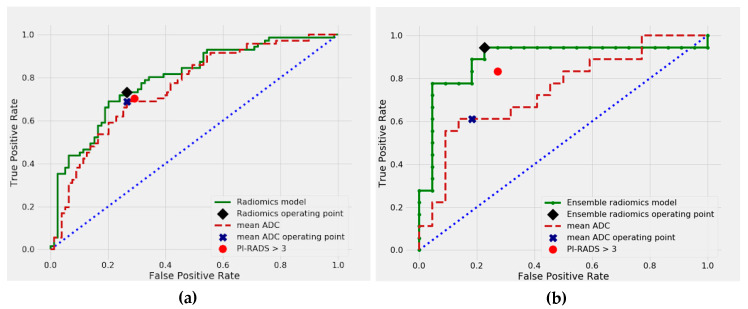
Receiver operating characteristics (ROC) curves for malignant vs. benign prostate lesion classification, in (**a**) 5-fold cross-validation, and (**b**) independent test set.

**Figure 2:**
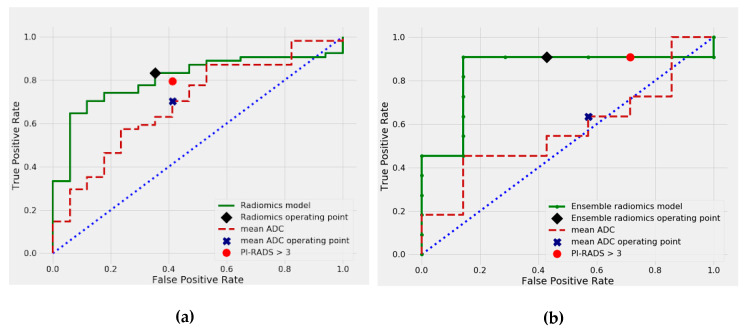
ROC curves for csPCa vs. cisPCa classification, in (**a**) cross-validation, (**b**) independent test set. csPCa = clinically significant prostate cancer; cisPCa = clinically insignificant prostate ca.ncer.

The combined results of our ROC curve analysis are presented in Table 2, with the training results calculated in the setting of repeated 5-fold cross-validation. Visual comparison of PI-RADS, mADC, and the selected models is shown in Figure 1 for the malignant vs. benign classification and in Figure 2 for the csPCa vs. cisPCa classification. Thresholds selected for sensitivity analysis corresponding to PI-RADS were 0.471 for radiomics and 1003 × 10^−6^ mm^2^/s for mADC for malignant vs. benign classification, and 0.45 for radiomics and 987 × 10^−6^ mm^2^/s for mADC for csPCa vs. cisPCa classification. 

### 2.2. Differentiation between Malignant and Benign Prostate Lesions

In the cross-validated analysis, the radiomics model achieved good predictive performance with a mean AUC of 0.783 for the differentiation of malignant vs. benign prostate lesions. In comparison, alternative clinical or imaging-based predictors numerically showed inferior predictive performance: PI-RADS (AUC = 0.758, *p* = 0.368), mADC (AUC = 0.754, *p* = 0.222), PSAD (AUC = 0.780, *p* = 0.714) and DRE (AUC = 0.617, *p* < 0.001) (Table 2). The cross-validated sensitivity of the radiomics model was 75% (53 of 71), and thus, marginally but not significantly higher than that of radiologist assessment using PI-RADS (70% (50 of 71), *p* = 0.832) or mean ADC values (69% (49 of 71), *p* = 0.581) (Table 3). In the independent test set, the radiomics model ensembled with PI-RADS, PSAD, and DRE achieved an excellent predictive performance with an AUC of 0.889, numerically higher than both PI-RADS (AUC = 0.779, *p* = 0.054) and mADC (AUC = 0.745, *p* = 0.067). The sensitivity of the ensemble radiomics model was 94% (17 of 18 lesions), compared with 83% for PI-RADS (15 of 18) (*p* = 0.5) and 61% for mADC (11 of 18) (*p* = 0.031), which proved that the model combining radiomics, PI-RADS and clinical predictors significantly improved sensitivity over mADC.

As a next step, we analyzed the subgroup of small index lesions, with volumes of less than 0.5 ml and compared it with the subgroup of large index lesions (volume ≥ 0.5 ml) [21,22]. This time, we performed ROC analysis for PCa detection only in the cross-validation cohort, to avoid small subgroup sample sizes in the test set (Table 4). In the small lesion subgroup, which included 71 patients, our radiomics model achieved a mean AUC of 0.678, comparable to PI-RADS (AUC = 0.694, *p* = 0.964) and mADC (AUC = 0.662, *p* = 0.574). In the large lesion subgroup (77 patients), our radiomics model with a mean AUC of 0.890 was numerically superior to the other two predictors, PI-RADS (AUC = 0.792, *p* = 0.093) and mADC (AUC = 0.829, *p* = 0.212), respectively.

### 2.3. Differentiation between csPCa and cisPCa

In the cross-validated analysis, the best performing radiomics model achieved a predictive AUC of 0.807, which was numerically higher than PI-RADS (AUC = 0.681, *p* = 0.144) and mADC (AUC = 0.697, *p* = 0.177) and showed a significantly better performance than PSAD (AUC = 0.644, *p* = 0.039) and DRE (AUC = 0.666, *p* = 0.039). The cross-validated sensitivity of the radiomics model was 83% (45 of 54). Within the training set, the sensitivity of the radiomics model was numerically but not significantly higher than that of radiologist assessment using PI-RADS (80% (43 of 54), *p* = 0.803) and mADC (70% (45 of 54), *p* = 0.092). In the independent test set, our ensemble radiomics model combining quantitative radiomic features, PI-RADS, and clinical predictors (PSAD and DRE) achieved a numerically superior predictive performance with an AUC of 0.844 in comparison to PI-RADS alone (AUC = 0.688, *p* = 0.209) and performed significantly better than mADC (AUC = 0.571, *p* = 0.022) for the prediction of clinically significant prostate cancer. The sensitivity of the ensemble radiomics model was 91% (10 of 11 lesions), equal to PI-RADS (15 of 18), and higher than mADC (64% (11 of 18)). It should be noted, however, that at 91% sensitivity, the ensemble radiomics model achieved specificity of 57% (4/7) vs. 28% (2/7) for PI-RADS.

As in the previous task, we performed ROC analysis of csPCa vs. cisPCa classification separately for small (<0.5 ml) and large (≥0.5 ml) index lesion subgroups in the cross-validation cohort. The small lesion subgroup was represented by 32 patients. The radiomics model achieved a cross-validated mean AUC of 0.708, almost similar to PI-RADS (AUC 0.707, *p* = 0.972) and mADC (AUC 0.731, *p* = 0.731). In the large lesion subgroup (39 patients), the radiomics model with a mean AUC of 0.873 was significantly better than PI-RADS (AUC 0.619, *p* = 0.03), whereas mADC achieved a mean AUC of 0.633 (*p* = 0.212).

All features selected for our radiomics signature for both tasks are enumerated in Supplementary Table S1. Table 5 shows the Spearman correlation coefficients between ISUP grade and first five radiomic features selected for the csPCa detection task. The features come from all three segmentation VOIs (ADC lesion, T2w lesion, and T2w whole gland masks) and are negatively correlated with ISUP grade. Two features extracted from the T2w images, one related to whole gland shape and one quantifying lesion homogeneity, are weakly correlated with high statistical significance (*p* < 0.05). 

Supplementary Table S2 presents model performance within each separate zone. It can be observed that for both tasks, our model is more accurate in classifying lesions located in the peripheral zone rather than the transition zone. 

## 3. Discussion

We investigated whether a machine learning model developed with quantitative radiomic features extracted from pretreatment mpMRI of the prostate could successfully differentiate between (i) malignant and benign prostate lesions and (ii) csPCa and cisPCa. Such a model could prospectively improve the clinical selection process for prostate biopsy and potentially tailor individual treatment decisions. 

The main finding of our study is that certain radiomic features extracted from mpMRI are reliable quantitative imaging biomarkers for PCa detection and classification and machine learning models using them show good predictive performance in comparison to mADC and PI-RADS as well as clinical biomarkers such as PSAD and DRE. Furthermore, our ensemble model combining radiomics, PI-RADS, PSAD, and DRE resulted in a predictive performance numerically superior to PI-RADS with regard to ROC and sensitivity analyses for both considered classification tasks. In detail, our ensemble model achieved a high predictive AUC of 0.889 for PCa detection and an AUC of 0.844 for csPCa vs. cisPCa classification in the independent test set. As a comparison, PI-RADS-based assessment resulted in AUC of 0.779 (*p* = 0.054) and 0.688 (*p* = 0.209), for cancer detection and csPCa classification, respectively. The ensemble model showed a significantly better predictive performance for csPCa vs. cisPCa classification than mADC, as an established quantitative imaging biomarker (AUC = 0.571; *p* = 0.035).

Several previous studies outlined the potential of radiomics for PCa characterization in mpMR images. Antonelli et al. [18] found that zone-specific models combining PSAD and radiomic features outperformed experienced radiologist assessments for the csPCa detection. Chaddad et al. [23] found a significant correlation between GLCM features extracted jointly from T2w and ADC images and Gleason score, with an AUC of 0.78 for GS ≤ 3, 0.82 for GS 3+4 and 0.65 for GS ≥ 4+3. Monti et al. [24] found that for PCa detection, standard radiomics models using T2w and ADC images performed better than an advanced model with additional diffusion kurtosis imaging and DCE. Approaches extracting radiomic features from prostate mpMRI using auto-fixed VOIs have also achieved promising results on peripheral zone csPCa detection, with the highest AUC of 0.87 for the XGBoost classifier [19]. On the other hand, Bonekamp et al. [20] showed that for PCa detection, mADC alone achieved good results, comparable to clinical interpretation, and found no further benefit with complex radiomics and machine learning methods, which is in line with our study results. Recent approaches using deep neural networks for lesion classification have also been reported to perform comparably to PI-RADS for PCa characterization using mpMRI [17,25]. However, their application in clinical routine is currently limited by poor generalization and low model interpretability.

A prior study by Hötker et al. [26] found a good to moderate AUC of 0.693 for mean ADC values in a dataset of 158 patients to detect csPCa. This is in line with the results of our study, where the predictive power of mADC measured with AUC varied from 0.697 to 0.754, depending on the task. Despite good repeatability, mean ADC values were less discriminative than either PI-RADS or our ensemble radiomics model and were achieving lower AUC for csPCa detection (*p* = 0.022) as well as lower sensitivity for PCa detection (*p* = 0.031) in comparison to our ensemble radiomics model. It is worth noting that the mRMR algorithm preferred the median ADC value as a more predictive feature than mADC in our cohort. We also found that other radiomic features, associated with shape and texture in T2w images, were correlated with Gleason scoring (Table 5).

Our study included a considerable number of small lesions (volume < 0.5 ml) with 23/54 and 5/11 ISUP grade ≥ 2 tumors in the training and test cohort, respectively. This threshold was first suggested by Epstein et al. [21] to separate insignificant from moderate/advanced tumors based on an earlier study by Stamey et al. [22], who outlined that the majority of prostate cancers smaller than 0.5ml are not likely to reach clinical significance. Since then, multiple recent studies confirmed that low risk prostate cancer is generally a low volume disease and a cut-off of 0.5 ml is commonly used to distinguish insignificant from clinically significant cancer [15,27,28]. Smaller index lesions are often challenging for visual analysis in daily clinical routine and may be overlooked, potentially resulting in lower cancer detection rates. We analyzed this subgroup and found that radiomics achieved a cross-validated AUC of 0.678 for task (i) and an AUC of 0.686 for task (ii), compared with AUCs of 0.890 and 0.873, respectively, in the subgroup of large lesions (≥0.5ml). In the small lesion subgroup, PI-RADS was non-inferior to our model, which suggests that radiologist reassessment could measurably improve the predictions. Previous studies commonly excluded lesions with volumes of less than 0.5 ml from their analysis [19,26,29,30,31,32] or provided no information about the distribution of lesion volumes [20,33].

Our radiomics model detects and classifies prostate cancer with higher accuracy in the peripheral zone than the transition zone. This is in agreement with recent reports showing a better performance of diffusion-weighted imaging for peripheral zone lesion characterization [8]. Although the advantage of quantitative measures (over qualitative clinical assessment) was also shown for the transition zone [34], the limitation of quantitatively assessing prostate cancer in the transition zone is related to overlapping features for cancers and nodules due to stromal benign prostatic hyperplasia [35]. This is also reflected within the zone-specific assessment of the PI-RADS classification and its modifications during recent years [8].

We found that the addition of clinical parameters, including DRE and PSAD to the radiomics model yielded predictive performances superior to PI-RADS, in regard of AUC and sensitivity/specificity for both considered tasks, although the differences did not achieve statistical significance in the majority of comparisons. However, in our test set, the ensemble model incorporating PI-RADS and clinical predictors (PSAD, DRE) did show an excellent predictive performance which was significantly superior to mADC, an established quantitative imaging biomarker for clinical significance prediction. Our results support the idea of an MRI-guided pathway for treatment planning [36] and underline the potential of novel imaging-based radiomics and clinical biomarkers for patient risk stratification. Automatic machine learning assessment of the prostate and characterization of suspicious index lesions in MRI could provide more comprehensive imaging biomarkers for improved cancer detection and risk stratification. 

Our study has a number of potential limitations. First of all, the study is only of a medium sample size. A larger patient cohort could improve the stability (especially for the subgroup analysis of small prostate lesions) and enable a more precise estimation of population statistics. Secondly, no external validation cohort was available for our study. Finally, we based the radiomics analysis on a biparametric MRI protocol, while radiologists additionally use DCE MRI in daily clinical routine as recommended in PI-RADS. Including other sequences in the analysis could potentially improve our results in larger prospective studies.

## 4. Materials and Methods 

### 4.1. Patient Cohort

**Figure 3:**
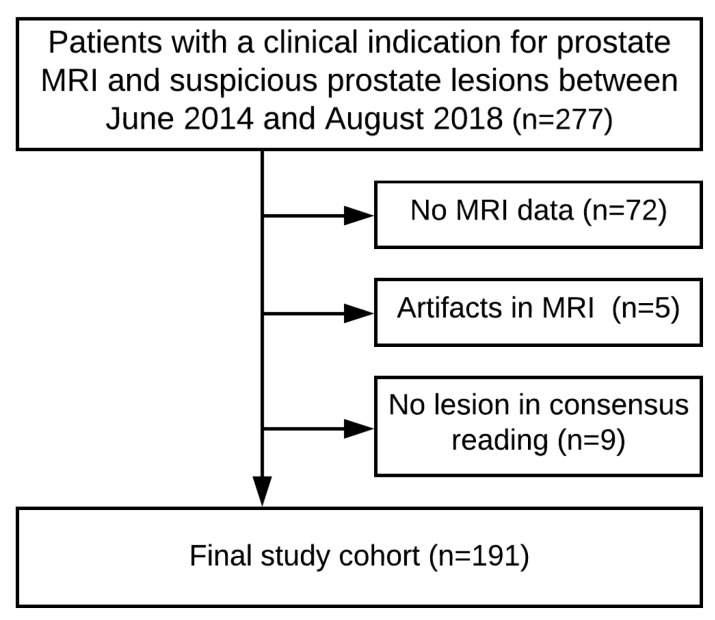
Consort flow diagram for patient inclusion.

Our retrospective single-center study was approved by the ethics committee of Heidelberg University, Medical Faculty Mannheim (approval no.: 2018-846R-MA). For this study, a database of 277 patients with a clinical indication of mpMRI from June 2014 to September 2018 was reviewed. Inclusion criteria were: (a) clinical suspicion of prostate cancer, (b) mpMRI of the prostate performed in our institution, and (c) systematic and targeted MRI/-ultrasound fusion biopsy performed within 3 months of an MRI exam. Exclusion criteria were: (a) unavailability of MRI data, (b) limited image quality, or (c) no visible index lesion on MRI. A study flow chart reflecting the exclusion criteria of patients can be found in Figure 3. Of the 277 enrolled patients, 205 had an available mpMRI of the prostate. Of those, 5 patients had to be excluded due to limited image quality or motion artifacts. A further 9 patients had to be excluded due to lack of an index lesion in consensus MRI reading. The final study cohort comprised 191 men with a clinical indication for prostate MRI and suspicious prostate lesions.

### 4.2. MRI Data Acquisition

All mpMR images were acquired on two different in-house 3-Tesla MRI scanners (Siemens Magnetom Skyra and Trio, Erlangen, Germany) with pelvic phased array coils. Details of the institutional mpMRI protocols are described in Supplementary Table S3. Biparametric MRI sequences consisting of T2-weighted and DWI/ADC-imaging were subjected to further analysis. MpMR images were quantitatively assessed by a board-certified radiologist (D.N. with 7 years of experience in prostate cancer imaging) and board-certified urologist (N.W. with 7 years of experience in biopsy acquisition) in accordance with PI-RADS classification standards. Cases with unclear lesions and/or PI-RADS scores were reviewed in consensus. We classified lesions in the central zone and the anterior fibromuscular stroma as transition zone lesions according to the PI-RADS recommendations and treated them as such in subsequent analysis. For further analysis, we dichotomized PI-RADS by considering radiologist evaluations as positive when the assigned PI-RADS score was 4 or 5, i.e., csPCa likely or highly likely.

Histopathologic findings from targeted prostate biopsy provided the standard of reference for the presence and clinical significance of prostate cancer using an index lesion-based approach. MRI lesions considered suspicious for malignancy were sampled by targeted MRI/transrectal ultrasound (TRUS) fusion biopsies followed by a systematic 12-core TRUS biopsy. Biopsy specimens were further processed and analyzed by local expert uropathologists according to consensus recommendations.

### 4.3. Image Segmentations

**Figure 4:**
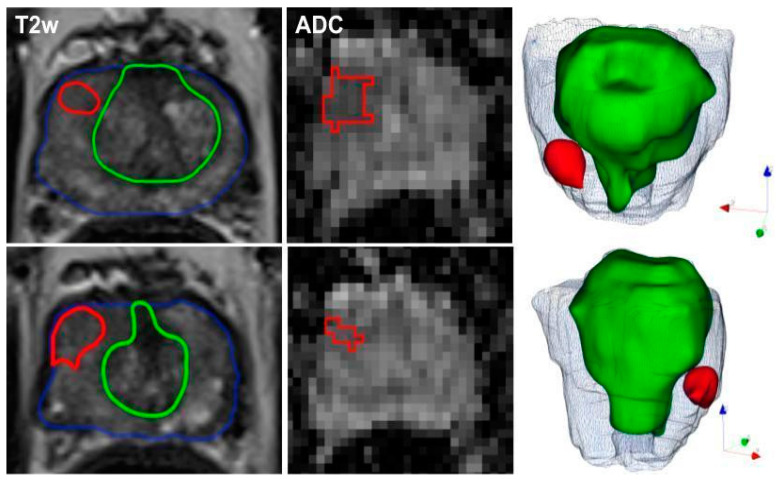
Representative segmentation of a 60-year-old man on axial T2-weighted and ADC-images with an initial prostate-specific antigen level of 12.9 ng/ml, normal DRE result, and a highly suspicious lesion located medio-apically in the right peripheral zone (PI-RADS category 4). Targeted MRI/ultrasound-fusion biopsy confirmed the presence of PCa, Gleason 3+4, ISUP 2. The contours outline the following manual segmentations: blue—whole organ, green—transition zone, red—index lesion.

For each patient, the whole prostate gland, peripheral and transition zones, as well as index lesions of the prostate were manually segmented on T2-weighted images. Additionally, prostate lesions were segmented separately on ADC images. Manual segmentations were performed by a medical student (P.W., 2 years of experience in prostate mpMRI) using the Medical Imaging Interaction Toolkit (MITK) and subsequently, reviewed and corrected in consensus by a board-certified radiologist (D.N.). The three-dimensional volumes of interest (VOIs) were manually drawn on consecutive axial slices. For patients with multiple tumor foci, only the index lesion (with the highest PI-RADS score or the biggest lesion volume) was selected for analysis. All segmentations were performed blinded to the PI-RADS score, clinical data, and histopathologic results. Figure 4 shows a representative PCa patient with manual segmentations of the whole prostate, transition zone, and a segmented lesion in the right peripheral zone (right apex), which was scored as PI-RADS4.

### 4.4. Radiomic Feature Extraction

Radiomic features were extracted using pyradiomics v3.0 [37], an open-source python package. We extracted features from the volume of interest (VOI) derived from T2w and ADC images and additionally, from the whole organ VOI in T2w to include more context. Three alternative feature setups were compared, including all available feature classes (shape, first order, GLRLM, GLSZM, and GLDM, in total, 232 features), as well as the features suggested by Schwier et al. as most repeatable in prostate T2w images (in total, 90 features) [33] and feature classes suggested by a radiologist of our institute (DN) with substantial experience in the field of radiomics (in total, 45 features). Supplementary Table S4 describes the exact feature classes used for each setup. Furthermore, we employed the Maximum Relevance Minimum Redundancy (mRMR) algorithm [38] to select a subset of the 15 most predictive and least correlated features.

### 4.5. Model Development

Machine learning models were developed and evaluated on the training dataset in the setting of a repeated 5-fold cross-validation with bootstrap bias correction (BBC-CV), which allows for both (i) selection of the optimal model and (ii) unbiased estimation of its performance [39]. Four methods were compared, namely logistic regression (LR), linear support vector machine (SVM), random forest (RF), and XGBoost. Each of them performed classification based on the priorly extracted radiomic features. The models were computed using scikit-learn (http://scikit-learn.org/stable/index.html). Convolutional neural networks (CNN), which used images cropped to the lesion region, were also included in the comparison. We followed the method described by Liu et al. [25] with T2w and ADC sequences concatenated as input. Hyperparameter tuning and the model selection process within cross-validation are described in the Supplementary Materials (Appendix A.1 and Table S5).

The radiomics model was compared with radiologist assessments represented by PI-RADS, quantitative mADC values from diffusion-weighted imaging as well as clinical predictors including PSAD and DRE within BBC-CV. Receiver operating characteristic (ROC) curves, as well as sensitivities and specificities, were calculated. We selected the radiomics model, which performed best in the cross-validation and created an ensemble classifier combining this model with logistic regression models for PI-RADS, PSAD, and DRE by majority voting. The ensemble was evaluated on an independent test cohort. 

To address the problem of class imbalance, we created synthetic data for the minority class with the adaptive synthetic (ADASYN) sampling approach [40], which reduces bias from class imbalance and shifts the decision boundary toward the difficult cases.

### 4.6. Statistical Analysis

The performance of the machine learning model developed with radiomic features was compared with the quantitative measurement of mADC, PI-RADS, PSAD, and DRE using the area (AUC) under the receiver operating characteristic (ROC) curve. Age, PSA as well as prostate and lesion volumes were checked for normality using the Shapiro-Wilk test. Univariable statistics are reported by p-value determined via student’s t-test if applicable (Shapiro-Wilk and Levene test) or Wilcoxon’s rank-sum test for continuous variables. A two-sided *p*-value < 0.05 was considered significant. The Spearman correlation coefficient (ρ) was calculated to establish the correlation strength of the features used in the radiomics model with ISUP grade. 

ROC curves were generated for each independent variable using bootstrap and compared using the DeLong test [41]. Cut-off values for the machine learning model and mADC were selected to match the specificity of the radiologist in the training dataset, so as to select operating points with low false positive rate. The sensitivity and specificity were compared for the machine learning model, quantitative measurement of mADC, and PI-RADS utilizing the McNemar test [42]. The Holm-Bonferroni method was applied to correct for multiple comparisons. Statistical analyses were implemented in the programming language Python (version 3.6.8, Python Software Foundation, Wilmington, Delaware, USA).

## 5. Conclusions

We showed that radiomics, using quantitative image data, is able to accurately characterize index lesions of the prostate derived from mpMRI and performs comparably to radiologists for the differentiation of (i) malignant from benign prostate lesions and (ii) csPCa from cisPCa. Machine learning models developed with quantitative radiomic features extracted from mpMRI represent a potential imaging biomarker, which, when combined with PI-RADS, PSAD, and DRE, predicts csPCa more accurately than mADC. Prognostic machine learning models could assist in csPCa detection and selection of patients for MRI-guided fusion biopsies. Further prospective, multicentric studies are needed to evaluate the clinical performance of machine learning models as a supporting tool for radiologists to improve MRI-directed therapy planning and risk stratification of patients on an individual level.

## Figures and Tables

**Table 1:** Demographic and clinical characteristics as well as MRI findings of all 191 enrolled patients.

Variable	Training Set	Test Set
Patients in total	151	40
PCa-negative	80	22
PCa-positive:	71	18
ISUP grade 1 (GS = 6)	17 (24%)	7 (39%)
ISUP grade ≥ 2 (GS ≥ 7)	54 (76%)	11 (61%)
ISUP grade 2	26 (37%)	3 (17%)
ISUP grade 3	14 (20%)	5 (28%)
ISUP grade 4	11 (15%)	3 (17%)
ISUP grade 5	3 (4%)	−
Zonal distribution of index lesions:		
Peripheral zone	102 (67.55%)	28 (70%)
Transition zone	49 (32.45%)	12 (30%)
Median age (years)	68 (63–74)	69 (63–72)
Median PSA value (ng/ml)	7.60 (5.71–11.00)	8.17 (6.82–11.85)
Median PSA density (ng/ml^2^)	0.161 (0.108–0.241)	0.194 (0.134–0.291)
Median prostate volume (ml)	48.4 (33.4–67.9)	42.6 (30.2–67.7)
Median lesion volume (ml)	0.53 (0.34–0.91)	0.53 (0.32–0.98)
MRI index lesion evaluation:		
PI-RADS 2	8 (5%)	5 (12%)
PI-RADS 3	70 (46%)	14 (35%)
PI-RADS 4	43 (28%)	14 (35%)
PI-RADS 5	30 (20%)	7 (23%)
Prior biopsy status:		
No biopsy	73	13
Prior biopsy negative	59	22
Prior biopsy positive	19	5
Prior transurethral resection of the prostate (TURP)	17	5

* in square brackets interquartile range: 25% value-75% value. PSA = Prostate specific antigen; PCa = Prostate cancer; ISUP = International Society of Urological Pathology; GS = Gleason score.

**Table 2:** Results of ROC analysis for the two selected classification tasks.

Predictor	Malignant vs. Benign Lesions			csPCa vs. cisPCa		
Cohort	mean AUC	95% CI *	*p-Value* ^♱^	mean AUC	95% CI *	*p-Value* ^♱^
Training						
PI-RADS	0.758	(0.671–0.817)	0.368	0.681	(0.572–0.786)	0.144
mADC	0.754	(0.677–0.827)	0.222	0.697	(0.592–0.781)	0.177
PSAD	0.780	(0.704–0.859)	0.714	0.644	(0.545–0.740)	**0.039** **^⤲^**
DRE	0.617	(0.556–0.667)	**<0.001** **^⤲^**	0.666	(0.605–0.721)	**0.039** **^⤲^**
**Radiomics model**	**0.783**	(0.682–0.875)	ref.	**0.807**	(0.691–0.906)	ref.
Test						
PI-RADS	0.779	(0.603–0.922)	0.054	0.688	(0.431–0.889)	0.209
mADC	0.745	(0.583–0.887)	0.067	0.571	(0.277–0.691)	**0.022** **^⤲^**
**Ensemble radiomics model**	**0.889**	(0.751–0.990)	ref.	**0.844**	(0.6–1.0)	ref.

* 95% confidence interval was calculated with BBC-CV for the radiomics model and with bootstrapping for the other variables. ^♱^ calculated with DeLong test for differences in AUC ROC compared with the reference (= ref.). ^⤲^ statistically significant difference. BBC-CV = Bootstrap Bias Corrected Cross Validation.

**Table 3:** Diagnostic performance of radiologist interpretation and radiomics on a per patient basis.

Predictor	Sensitivity (%) *	95% CI (%)	*p* Value ^♱^	Specificity (%) *	95% CI (%)
Cohort	**malignant vs. benign lesions**				
Training					
PI-RADS	70 (50/71)	(59–81)	0.832	71 (56/79)	(60–81)
mADC	69 (49/71)	(56–77)	0.581	73 (58/79)	(65–84)
Radiomics model	75 (53/71)	(57–87)	reference	73 (58/79)	(56–83)
Test					
PI-RADS	83 (15/18)	(74–91)	0.500	73 (16/22)	(63–82)
mADC	61 (11/18)	(50–73)	0.031	82 (18/22)	(73–90)
Ensemble radiomics	94 (17/18)	(88–99)	reference	77 (17/22)	(68–86)
	**csPCa vs. cisPCa**				
Training					
PI-RADS	80 (43/54)	(69–90)	0.803	59 (10/17)	(36–83)
mADC	70 (38/54)	(58–81)	0.092	59 (10/17)	(33–82)
Radiomics model	83 (45/54)	(69–95)	reference	65 (11/17)	(20–100)
Test					
PI-RADS	91 (10/11)	(82–98)	1.0	28 (2/7)	(13–46)
mADC	64 (7/11)	(50–78)	0.250	43 (3/7)	(24–60)
Ensemble radiomics	91 (10/11)	(81–98)	reference	57 (4/7)	(38–74)

* in brackets proportion of raw data. ^♱^ McNemar test for differences in sensitivity compared with reference. csPCa = clinically significant prostate cancer; cisPCa = clinically insignificant prostate cancer.

**Table 4:** Analysis of patient subgroups with small (<0.5 ml) and large (≥0.5 ml) index lesions in the cross-validation cohort.

Cohort *	Predictor	Mean AUC	95% CI	p-Value ^♱^
**malignant vs. benign lesions**				
Small lesions (71)	PI-RADS mADC Radiomics model	0.694 0.662 0.678	(0.582–0.803) (0.530–0.781) (0.560–0.814)	0.964 0.574 reference
Large lesions (77)	PI-RADS mADC Radiomics model	0.792 0.829 0.890	(0.694–0.882) (0.736–0.915) (0.812–0.953)	0.093 0.212 reference
**csPCa vs. cisPCa**				
Small lesions (32)	PI-RADS mADC Radiomics model	0.707 0.731 0.708	(0.506–0.875) (0.537–0.897)v(0.525–0.880)	0.972 0.801 reference
Large lesions (39)	PI-RADS mADC Radiomics model	0.619 0.633 0.873	(0.385–0.853) (0.388–0.838) (0.701–0.993)	**0.030** 0.086 reference

* in brackets raw number of patients. ♱ DeLong test for differences in AUC ROC compared with the reference. csPCa = clinically significant prostate cancer; cisPCa = clinically insignificant prostate cancer.

**Table 5:** Spearman correlation coefficient (ρ) for correlation of the ISUP grade with the first five radiomic features chosen by mRMR for csPCa vs. cisPCa prediction.

Feature	Sequence	VOI	*ρ*	*p*-Value
‘original_shape_Maximum2DDiameterColumn’	T2	whole gland	−0.229	0.031 ^⤲^
‘original_firstorder_Maximum’	ADC	lesion	−0.110	0.305
‘original_shape_Sphericity’	T2	whole gland	−0.154	0.149
‘original_glrlm_GrayLevelNonUniformityNormalized’	T2	lesion	−0.260	0.014 ^⤲^
‘original_shape_Elongation’	ADC	lesion	−0.11	0.301

^⤲^ statistically significant correlation. ISUP = International Society of Urological Pathology; csPCa = clinically significant prostate cancer; cisPCa = clinically insignificant prostate cancer.

## Supplementary Materials

The following are available online at https://www.mdpi.com/2072-6694/12/7/1767/s1. Table S1: All selected features for our radiomics signature, Table S2: Zonal subgroup analysis of the diagnostic performance of the radiomics model, PI-RADS and mADC, Table S3: Multiparametric prostate MRI protocol, Table S4: Radiomics feature classes used for each setup. Table S5: Model selection on the training set.

## Appendix A

### Appendix A.1. Details of Hyperparameter Tuning and Model Selection Process

We performed hyperparameter optimization in order to select the best configuration for each method (LR, SVM, RF, and XGBoost) in terms of AUC, using Grid Search. The search was integrated into the repeated 5-fold cross-validation with bootstrap bias correction and used bootstrap sampling of the pooled predictions from all the 5 folds, separately for each task. The best configurations for each method are presented in Table S5. We finally chose the optimized model with the highest AUC among included models to be used in the ensemble model, which was evaluated on an independent test set.

It is worth noting that statistics for the training cohort were not calculated for the best-performing model. For every repetition, the BBC-CV procedure bootstrapped the strategy for selecting the best configuration and mean AUC was calculated on the samples not selected by the bootstrap (more details in [39]).
