# Supplementary material for: Multiparametric MRI for Prostate Cancer Characterization: Combined Use of Radiomics Model with PI-RADS and Clinical Parameters
Source: Cancers (Basel). 2020 Jul 2;12(7):1767. doi: 10.3390/cancers12071767 (PMC7407326; doi:10.3390/cancers12071767)
Supplement: Supplementary file 1 [file cancers-12-01767-s001.pdf]

cancers

MDPI

Table S1. All selected features for our radiomics signature.

Feature	MRI sequence	VOI
**Malignant vs. benign lesions**		
'original_firstorder_Median'	ADC	lesion
'original_glrlm_RunLengthNonUniformity'	T2	wholegland
'original_shape_Flatness'	T2	whole gland
'original_glrlm_ShortRunLowGrayLevelEmphasis'	T2	whole gland
'original_shape_Sphericity'	T2	whole gland
'original_firstorder_Variance'	ADC	lesion
'original_shape_Flatness'	T2	lesion
'original_firstorder_10Percentile'	ADC	lesion
'original_shape_Maximum2DDiameterRow'	T2	whole gland
'original_firstorder_Energy'	ADC	lesion
'original_firstorder_Skewness'	ADC	lesion
'original_shape_Elongation'	T2	whole gland
'original_firstorder_Maximum'	ADC	lesion
'original_glrlm_LongRunEmphasis'	T2	whole gland
'original_shape_Elongation'	ADC	lesion
**csPCa vs. cisPCa**		
'original_shape_Maximum2DDiameterColumn'	T2	whole gland
'original_firstorder_Maximum'	ADC	lesion
'original_shape_Sphericity'	T2	whole gland
'original_glrlm_GrayLevelNonUniformityNormalized'	T2	lesion
'original_shape_Elongation'	ADC	lesion
'original_glrlm_LongRunHighGrayLevelEmphasis'	T2	lesion
'original_firstorder_Median'	ADC	lesion
'original_glrlm_ShortRunLowGrayLevelEmphasis'	T2	whole gland
'original_shape_Elongation'	T2	whole gland
'original_shape_Sphericity'	ADC	lesion
'original_firstorder_Skewness'	ADC	lesion
'original_glrlm_RunEntropy'	T2	whole gland
'original_shape_SurfaceVolumeRatio'	T2	whole gland
'original_shape_Flatness'	T2	lesionv
'original_firstorder_Kurtosis'	ADC	lesion

Table S2. Zonal subgroup analysis of the diagnostic performance of the radiomics model, PI-RADS and mADC.

Predictor	AUC ROC	95% CI	P value*	Sensitivity (%)°	95% CI (%)	*P* value†	Specificity (%)°	95% CI (%)
	**malignant vs. benign lesions**							
**Peripheral zone**								
PI-RADS	0.781	[0.694-0.856]	0.469	73 (38/52)	[60-85]	0.301	71 (35/49)	[58-84]
mADC	0.820	[0.730-0.898]	0.901	71 (37/52)	[58-83]	0.109	82 (40/49)	[80-92]
Radiomics model	0.824	[0.731-0.902]	ref.	83 (43/52)	[71-92]	ref.	75 (37/49)	[63-86]
**Transition zone**								
PI-RADS	0.683	[0.544-0.816]	0.979	63 (12/19)	[42-84]	0.453	70 (21/30)	[52-86]
mADC	0.601	[0.434-0.761]	0.302	63 (12/19)	[42-84]	0.25	60 (18/30)	[41-78]
Radiomics model	0.683	[0.544-0.817]	ref.	47 (9/19)	[25-70]	ref.	70 (21/30)	[52-86]
	**csPCa vs. cisPCa**							
**Peripheral zone**								
PI-RADS	0.660	[0.472-0.873]	0.040	80 (33/41)	[67-92]	0.581	54 (6/11)	[25-83]
mADC	0.809	[0.637-0.930]	0.284	76 (31/41)	[62-88]	0.125	73 (8/11)	[44-100]
Radiomics model	0.894	[0.801-0.971]	ref.	88 (36/41)	[76-97]	ref.	64 (7/11)	[33-91]
**Transition zone**								
PI-RADS	0.700	[0.371-0.910]	0.329	77 (10/13)	[53-100]	1.0	66 (4/6)	[25-100]
mADC	0.397	[0.092-0.641]	0.388	54 (7/13)	[29-82]	0.688	33 (2/6)	[0-75]
Radiomics model	0.587	[0.275-0.857]	ref.	69 (9/13)	[42-92]	ref.	67 (4/6)	[25-100]

*DeLong test for differences in AUC ROC compared with the reference

°in brackets proportion of raw data

†McNemar test for differences in sensitivity compared with reference.

Table S3. Multiparametric prostate MRI protocol.

MRI sequences	Orientation	TE (ms)	TR (ms)	Slice thickness (mm)	FoV read (mm)
**MRI scanner no. 1 (Siemens, Magnetom Trio, 3T)**					
DWI b50/500/800	tra	88	5000	3	204
t2_haste_sag	sag	105	1200	4	281*300
t2_tse_cor	cor	101	4000	3	200
t2_tse_tra	tra	101	4580	3	200
t2_tse_sag	sag	101	4190	3	200
t1_vibe_cor_iso	cor	2.15	4.71	0.91	290
t1_tse_tra	tra	9.8	858	3	200
**MRI scanner no. 2 (Siemens, Magnetom Skyra, 3T)**					
DCE (Grasp)	tra	1.69	3.85	1.5	240
DWI zoomit_epi	tra	69	3800	3	380
DWI zoomit_epi_2000	tra	91	4600	3	380
t1_twist_tra	tra	1.97	5.05	3.5	260
t2_tse_tra	tra	104	3300	3	200
t2_tse_sag	sag	104	3600	3	200
t2_tse_cor	cor	104	3200	3	200
DWI ep2d_dif	tra	69	3600	4	200

Table S4. Radiomic feature classes used for each setup.

Feature setup	Classes extracted from lesion VOI	Classes extracted from whole prostate VOI
All features	Shape<br>first order<br>GLRLM<br>GLSZM<br>GLDM	Shape<br>first order<br>GLRLM<br>GLSZM<br>GLDM
Features from Schwier et al. [1]	shape:<br>'VoxelVolume'<br>'SurfaceVolumeRatio'<br>'Maximum2DDiameterRow'<br>first order:<br>'Range'<br>'Minimum'<br>'90Percentile'<br>'Mean'<br>GLRLM:<br>'RunLengthNonUniformity'<br>'HighGrayLevelRunEmphasis'<br>'ShortRunHighGrayLevelEmphasis'<br>GLSZM:<br>'SmallAreaHighGrayLevelEmphasis'<br>'SizeZoneNonUniformityNormalized'<br>'HighGrayLevelZoneEmphasis'<br>GLCM:<br>'DifferenceAverage'<br>'Contrast'<br>'Autocorrelation'	shape:<br>'VoxelVolume'<br>'Maximum2DDiameterSlice'<br>'Maximum3DDiameter'<br>'Maximum2DDiameterColumn'<br>first order:<br>'Variance'<br>'RobustMeanAbsoluteDeviation'<br>'10Percentile'<br>GLRLM:<br>'RunPercentage'<br>'RunLengthNonUniformityNormalized'<br>'GrayLevelVariance'<br>GLSZM:<br>'ZoneEntropy'<br>'SmallAreaEmphasis'<br>'SizeZoneNonUniformityNormalized'<br>GLCM:<br>'JointEnergy'<br>'Imc1'<br>'Correlation'
Radiologist suggestion	shape<br>first order<br>GLRLM	shape<br>first order<br>GLRLM

Table S5. Model selection on the training set.

Model	Feature setup*	**mean AUC ROC** (100 repeats 5-fold CV)	
		malignant vs. benign	csPCa vs. cisPCa
**Radiomics:**			
Logistic Regression	All	0.785	0.721
	Custom	0.800	0.786
	VOI	0.796	0.638
SVM	All	0.783	0.738
	**Custom**	0.799	**0.814**
	VOI	0.783	0.660
Random Forest	All	0.798	0.646
	**Custom**	**0.804**	0.657
	VOI	0.779	0.570
XGBoost	All	0.797	0.594
	Custom	0.793	0.624
	VOI	0.747	0.572
**Deep learning:**			
EfficientNet	–	0.702	0.657
XMasNet	–	0.712	0.674

*Feature setup abbreviations: *All* - all features, *VOI* - features suggested by Schwier et al., *Custom* - radiologist suggestion

Reference

1. Schwier, M.; Griethuysen, J. V.; Vangel, M. G.; Pieper, S.; Peled, S.; Tempany, C.; Aerts, H. J. W. L.; Kikinis, R.; Fennessy, F. M.; Fedorov, A. Repeatability of Multiparametric Prostate MRI Radiomics Features. *Sci. Rep*. **2019**, *9*, 1–6.
